# Determining the viability of *Schistosoma mansoni* cercariae using fluorescence assays: An application for water treatment

**DOI:** 10.1371/journal.pntd.0008176

**Published:** 2020-03-26

**Authors:** Laura Braun, Lucinda Hazell, Alexander J. Webb, Fiona Allan, Aidan M. Emery, Michael R. Templeton

**Affiliations:** 1 Department of Civil and Environmental Engineering, Imperial College London, London, United Kingdom; 2 Section of Structural and Synthetic Biology, Department of Infectious Disease, Imperial College London, London, United Kingdom; 3 Wolfson Wellcome Biomedical Laboratories, Department of Life Sciences, Natural History Museum, London, United Kingdom; Wellcome Trust Sanger Institute, UNITED KINGDOM

## Abstract

**Background:**

Schistosome cercariae are the human-infectious stage of the *Schistosoma* parasite. They are shed by snail intermediate hosts living in freshwater, and penetrate the skin of the human host to develop into schistosomes, resulting in schistosomiasis infection. Water treatment (e.g. filtration or chlorination) is one way of cutting disease transmission; it kills or removes cercariae to provide safe water for people to use for activities such as bathing or laundry as an alternative to infested lakes or rivers. At present, there is no standard method for assessing the effectiveness of water treatment processes on cercariae. Examining cercarial movement under a microscope is the most common method, yet it is subjective and time-consuming. Hence, there is a need to develop and verify accurate, high-throughput assays for quantifying cercarial viability.

**Method:**

We tested two fluorescence assays for their ability to accurately determine cercarial viability in water samples, using *S. mansoni* cercariae released from infected snails in the Schistosomiasis Collection at the Natural History Museum, London. These assays consist of dual stains, namely a vital and non-vital dye; fluorescein diacetate (FDA) and Hoechst, and FDA and Propidium Iodide. We also compared the results of the fluorescence assays to the viability determined by microscopy.

**Conclusion:**

Both fluorescence assays can detect the viability of cercariae to an accuracy of at least 92.2% ± 6.3%. Comparing the assays to microscopy, no statistically significant difference was found between the method’s viability results. However, the fluorescence assays are less subjective and less time-consuming than microscopy, and therefore present a promising method for quantifying the viability of schistosome cercariae in water samples.

## Author summary

The role of water treatment in integrated interventions, alongside preventive chemotherapy and behavior change programs, is growing, as we now look towards elimination of schistosomiasis as a public health concern. So far, no guidelines have been developed for treating schistosome infested water. This is partially due to the lack of appropriate methods for measuring cercarial viability, which has impeded the research into the effectiveness of water treatment processes against schistosome cercariae. This study develops and validates two dual-staining, high-throughput fluorescence assays for quantifying cercarial viability in water samples. These methods will facilitate the testing of water treatment processes against cercariae, thereby bringing the knowledge of water treatment for schistosomiasis up to speed with the level of our knowledge of how to control other waterborne and water-based diseases. This will promote the development of water treatment guidelines and the design of effective water infrastructure for schistosomiasis-endemic regions.

## Introduction

Schistosomiasis is a water-based disease that leads to 200,000 deaths and 3.3 million disability-adjusted life years (DALYs) annually, primarily in Sub-Saharan Africa [1,2]. This parasitic disease is contracted through contact with cercaria-infested freshwater, such as rivers or lakes. Schistosome cercariae are up to 260 μm long and are released by their intermediate host, freshwater snails [3]. The cercariae penetrate human skin by secreting the enzyme elastase, and once inside the human host develop into adult worms [4]. These mate to produce eggs that exit from the human body in urine or feces. Miracidia hatch from the eggs when in contact with freshwater and complete the lifecycle by infecting snails. The disease can be controlled through preventative chemotherapy (by treating the primary host), behavior change and health education, universal access to sanitation (thus reducing the number of eggs entering freshwater bodies), snail control, and provision of safe water for water contact activities (e.g. bathing, laundry).

A recent review about the effectiveness of water treatment processes against schistosome cercariae identified the lack of protocols for rapidly and reliably quantifying cercarial viability [5]. This makes it difficult to assess the effectiveness of water treatment processes that aim to kill or remove cercariae in water.

Microscopy is the most common method for assessing cercarial viability, and involves observations of parasite movement and shape. Although often considered the “gold standard” [6], microscopy is subjective, time-consuming, and requires knowledge of parasite morphology [7,8]. Smart-microscopy methods using automated image analysis to assess parasite motility or morphological damage, and impedance-based real time cell platforms, have been developed for schistosomes and other parasites, and may prove useful for cercarial viability measurements [9–13].

Bioassays such as the skin attachment method (observing if cercariae can penetrate skin) or host-infection experiments (exposing animal hosts to cercariae, followed by worm perfusion to observe the resulting level of infection) can be used to assess larger samples at once. However, these methods are labor intensive, time-consuming, and require animal testing and therefore ethical approval [14,15].

Other techniques, such as colorimetry or fluorimetry, may enable a more reliable, ethical and quicker way to measure viability. These methods use vital and non-vital dyes that exclusively stain live or dead parasites. The change in the dye’s intensity can be used to quantify the sample’s viability (visually under a microscope, or automated using spectrophotometers, flow cytometers or plate readers) [8]. These methods have significant advantages in terms of standardization, accuracy, simplicity of experiments, and replicability [16]. Fluorescence and colorimetric assays for determining parasite viability have been reviewed by Keiser [15] and Peak and Hoffmann [8], and although some of these methods have been shown to work on part of the schistosome lifecycle (for example MTT formazan on adult worms [17,18] or methylene blue on schistosomula [19]), there is a need to develop high-throughput assays for detecting cercarial viability.

Here we adapt two fluorescence assays from previous schistosome viability studies [20,21] to identify if they can accurately determine cercarial viability. The two assays use a combination of vital and non-vital dyes that are widely available, have short incubation periods, and separate emission wavelengths: Hoechst 33258 (Hoechst), Propidium Iodide (PI) and Fluorescein Diacetate (FDA).

Hoechst is a cell-permeable dye that fluoresces blue when bound to DNA and is used as an indicator of membrane integrity, as fluorescence increases if cell membrane integrity is impaired [21]. PI is a non-vital, red fluorescent DNA stain, which is unable to cross intact cells and can only enter if there is a breach in the membrane [22,23]. FDA is a cell-permeable vital dye that is hydrolyzed to green fluorescein by the enzyme esterase [24]. The fluorescence is only retained in cells with intact membranes, thereby staining viable cells. These three dyes are used to double-stain cercariae, namely FDA and PI (FDA-PI) and FDA and Hoechst (FDA-H). The FDA-PI assay has been shown to accurately determine schistosomula viability by Peak et al. [20]. However, other researchers have struggled to replicate these results [6,16], and limitations of the assay have been found in other fields of research [24]. We therefore also test the FDA-H dual stain, since Hoechst has been used to determine cercarial viability [25]. Using a combination of vital and non-vital dyes, instead of using a single dye, may help increase the accuracy and validates that differences in dye uptake and fluorescence result from differences in viability [20].

## Materials and methods

### Cercaria preparation

*Schistosoma mansoni* infected *Biomphalaria glabrata* snails were obtained from the Biomedical Research Institute (Rockville, MD, USA) and kept at 27°C at the Natural History Museum (London, United Kingdom). Prior to experiments, 10 snails were placed in the dark for 48 hours to induce heavier shedding of the parasite larvae. The snails were then rinsed in bottled spring water (pH 7.2) and exposed to light (11W LED lamp) for 70 minutes. Snails were removed and the cercaria-infested water was filtered through a 200 μm polyester filter to remove snail feces. To estimate the number of cercariae (per 100 μl), three 50 μl aliquots were taken by pipette. 5 μl of Lugol iodine solution was added to kill and stain the cercariae, which could then be easily counted under a microscope. The cercaria solution was diluted with bottled spring water to achieve the desired parasite concentration (55 cercariae/100 μl). Unless otherwise stated, 100 cercariae were used per well in a 96-well plate. Based on aliquot data and applying a measure of uncertainty, cercaria numbers varied by ± 4 cercariae per well. Cercariae were aliquoted into black-sided, optically-clear, flat-bottomed, 96-well microtiter plates (ThermoFisher Scientific, catalog no M33089). For experiments involving non-viable samples, cercariae were heat-killed in a glass universal bottle placed in a 60°C water bath for 5 minutes, and allowed to cool to 27°C before being aliquoted into 96-well plates. For the mixed samples, 50% live and 50% dead cercariae were added together. Media (water without cercariae) were produced by passing cercaria-water through a 20 μm polyester filter. This removed cercariae, while allowing smaller particles and dissolved organic matter to pass. In those experiments that quantified viabilities, samples with the desired viability were produced by mixing the respective proportions of live and dead cercaria solutions (e.g. 25% viability was produced by mixing 75% heat-killed cercariae with 25% untreated cercariae). All experiments were run in triplicate and repeated at least three times.

### Dye preparation

Stock solutions of FDA (catalog no F7378, 0.025 mg/ml in acetone), PI (catalog no P4170, 0.02 mg/ml in 1 X phosphate buffered saline (PBS)) and Hoechst (catalog no 94403, 5 mg/ml in 1 X PBS) were stored in the dark at 4°C for up to 3 months. Stock solutions were added to cercaria samples to obtain final dye concentrations of 0.075, 0.5 and 2 μg/ml for Hoechst, FDA, and PI, respectively, based on previous literature [6,20,21]. All dyes were purchased from Sigma-Aldrich.

### Microscopy

Stained cercariae were examined under a Leica CTR 500 fluorescence microscope at 20Xmagnification equipped with filter cubes A, N2.1 and I3 for Hoechst, FDA and PI, respectively. Images were captured with a Leica DFC310 FX digital color camera and overlaid with the open-source GIMP software.

### Fluorescence measurements

Cercariae were aliquoted into 96-well plates in triplicate, and dyes (FDA-H, or FDA-PI) were added to obtain a final volume of 200 μl/well. The microplate was sealed with a polyester film and loaded into a BMG Labtech FLUOstar Optima plate reader. Fluorescence measurements were taken with an orbital scan diameter of 4 mm, at excitation and emission wavelengths of 355/460 nm (Hoechst), 485/520 nm (FDA), and 544/612 nm (PI). Plates were programmed to shake for 5 seconds at 150 rpm before each cycle (double orbital shaking mode) to promote even dispersion of cercariae. The temperature of the plate reader was set to 32°C to promote enzymatic hydrolysis of FDA, while at the same time not reducing the cercarial lifespan [20,26]. Wells were examined microscopically at the end of experiments and compared to a negative control (live cercaria sample left at room temperature) to confirm that the temperature and dyes did not affect cercarial viability.

### Data handling and statistical analysis

The cercarial viability was calculated using the equation below:

$$\%\;viability=\frac{alive}{dead}x100$$

*Alive* and *dead* represent the proportion of live and dead cercariae in the sample and are calculated with the following equations [20]:

$$Alive\;(using\;FDA\;fluroescence)=\frac{\left(sample-nonviable\;sample\right)}{\left(viable\;sample-nonviable\;sample\right)}$$

$$Dead\;(using\;PI\;or\;Hoechst\;fluorescence)=\frac{\left(sample-media\right)}{\left(nonviable\;sample-media\right)}$$

The *viable sample* and *non-viable sample* are the fluorescence (RFU) of untreated (100% viable) and heat-killed (0% viable) cercariae, respectively. Sample is the fluorescence (RFU) of the well whose viability we are determining, and *media* is the fluorescence (RFU) of the well containing only media (produced by filtering cercaria-water through a 20 μm filter). The mean and standard error of the mean (SEM) of the fluorescence data were calculated. A one-way analysis of variance (ANOVA) and post-hoc least significant difference test (p<0.05 and p<0.01) were used to determine if, and between which samples (live, mixed or dead), there was a statistically significant difference in fluorescence.

## Results

### Microscopy

**Fig 1:**
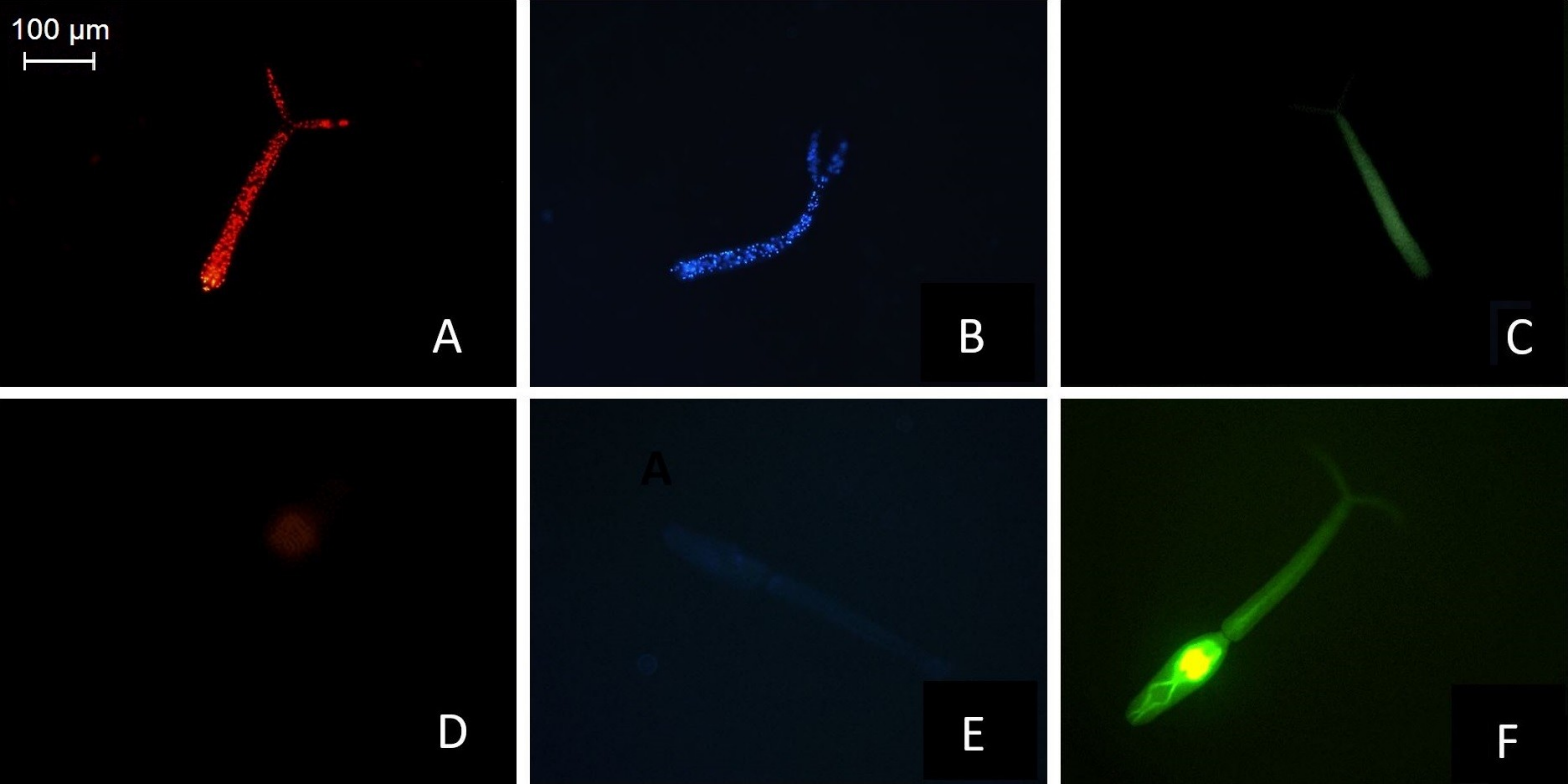
Individually PI (A and D), Hoechst (B and E) and FDA (C and F) stained cercariae. The PI and Hoechst-stained dead cercarial tails (A and B) show the staining of the nucleic acid. Both tails fluoresce strongest at the tail-head junction, as this is where most ruptured cell membranes are found resulting from the tail separation. The FDA-stained whole live cercaria (F) fluoresces strongest in the acetabular glands. Image D, E show a live cercaria stained with PI and Hoechst, respectively. Image C shows an FDA-stained dead cercaria.

**Fig 2:**
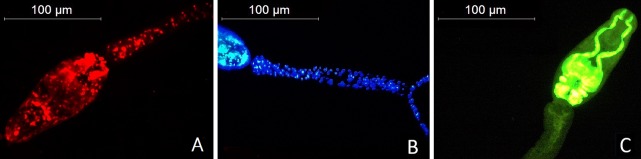
. Higher magnification images of PI (A), Hoechst (B) and FDA (C) stained cercariae. Dead cercariae are stained with PI and Hoechst, and a live cercaria stained with FDA.

Fluorescence microscopy was used to determine the dyes’ ability to independently label live or dead cercariae. To this end, 10 live and 10 dead cercariae were stained separately with the dyes, incubated for 10 minutes and examined under a microscope. All experiments were run in triplicate. The results show that 100% of live cercariae were FDA-stained, and 100% of dead cercariae were stained with PI or Hoechst (Fig 1F, 1A and 1B, respectively). The latter two dyes fluoresced primarily in the cell nuclei of dead cercariae, indicated by red (Fig 1A, Fig 2A) and blue (Fig 1B, Fig 2B) dots. Both PI and Hoechst-stained tails fluoresced strongest at the head-tail junction (Fig 1A and 1B), resulting from the tail shedding induced by the heat treatment. Cercariae are covered by a single unit membrane [27], and the head and tail are connected by a thin muscle layer that encloses the excretory vesicle which is ruptured during tail loss [28]. PI and Hoechst did not visibly stain live cercariae (Figs 1D, 1E, S1 and S2). FDA however did partially stain dead cercariae, though at a lower intensity than live cercariae (Fig 1C). As seen in Fig 1F and Fig 2C, FDA-fluorescence in live cercariae was brightest at the acetabular glands and dorsal to the acetabulum, where strong cellular activity is present [29].

**Fig 3:**
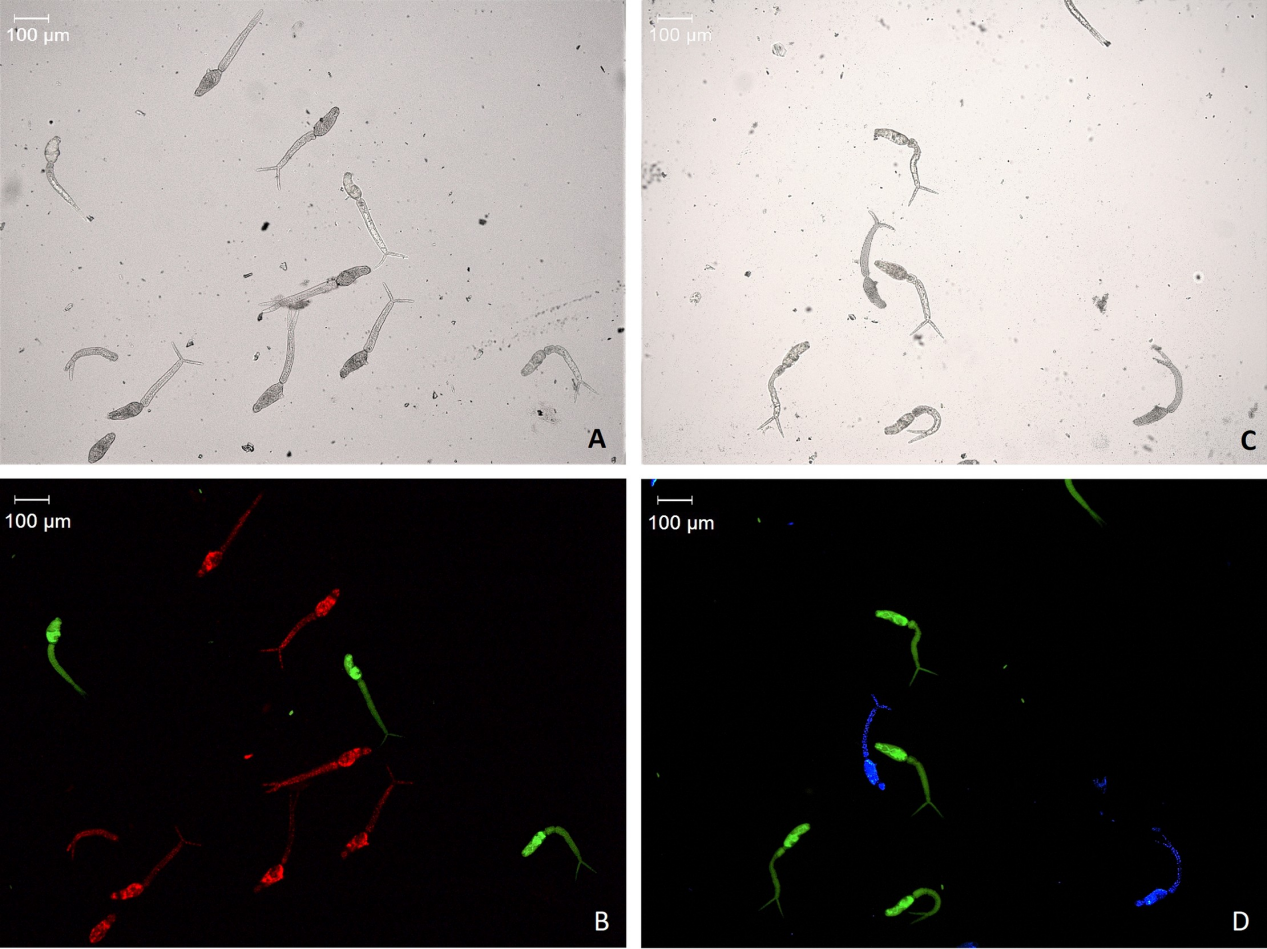
Cercariae double stained with FDA-PI (B) and FDA-H (D). Live, whole cercariae fluoresce green, whereas dead cercariae (often with their tail separated) fluoresce blue (Hoechst) or red (PI). Respective bright-field images of cercariae (A and C) shown for reference.

To assess whether combinations of vital and non-vital dyes will enable an accurate measurement of cercarial viability, a mixture of 10 live and 10 dead cercariae was stained with two fluorophores, in this case FDA-PI, and FDA-H. Stained cercariae were incubated for 10 minutes and examined under the microscope. Experiments were run in triplicate. The overlaid images shown in Fig 3B–3D confirm that FDA exclusively stained live cercariae, and PI and Hoechst dead cercariae. No cercariae fluoresced in both fluorophores (using either filter cubes), or in yellow or cyan, indicating that the dyes stain different areas of cells, and hence there is no co-localization [30]. These results are comparable to the non-cercaria studies of Yan *et al*. [31], Peak *et al*. [20] and Jiajia *et al*. [32], and therefore all three fluorophores are suitable for determining cercarial viability.

### Determination of incubation time using plate reader

To determine the optimal dye incubation time, fluorescence measurements of stained cercariae (100 ± 4 cercariae/well) were taken every 5 minutes for 60 minutes. Live, dead, a mix of live and dead cercariae, media, and a blank were examined at each time point. A short incubation time is desirable to minimize natural cercarial die-off, whilst ensuring that there is a statistically significant difference between the fluorescence of live, mixed, and dead cercariae.

**Fig 4:**
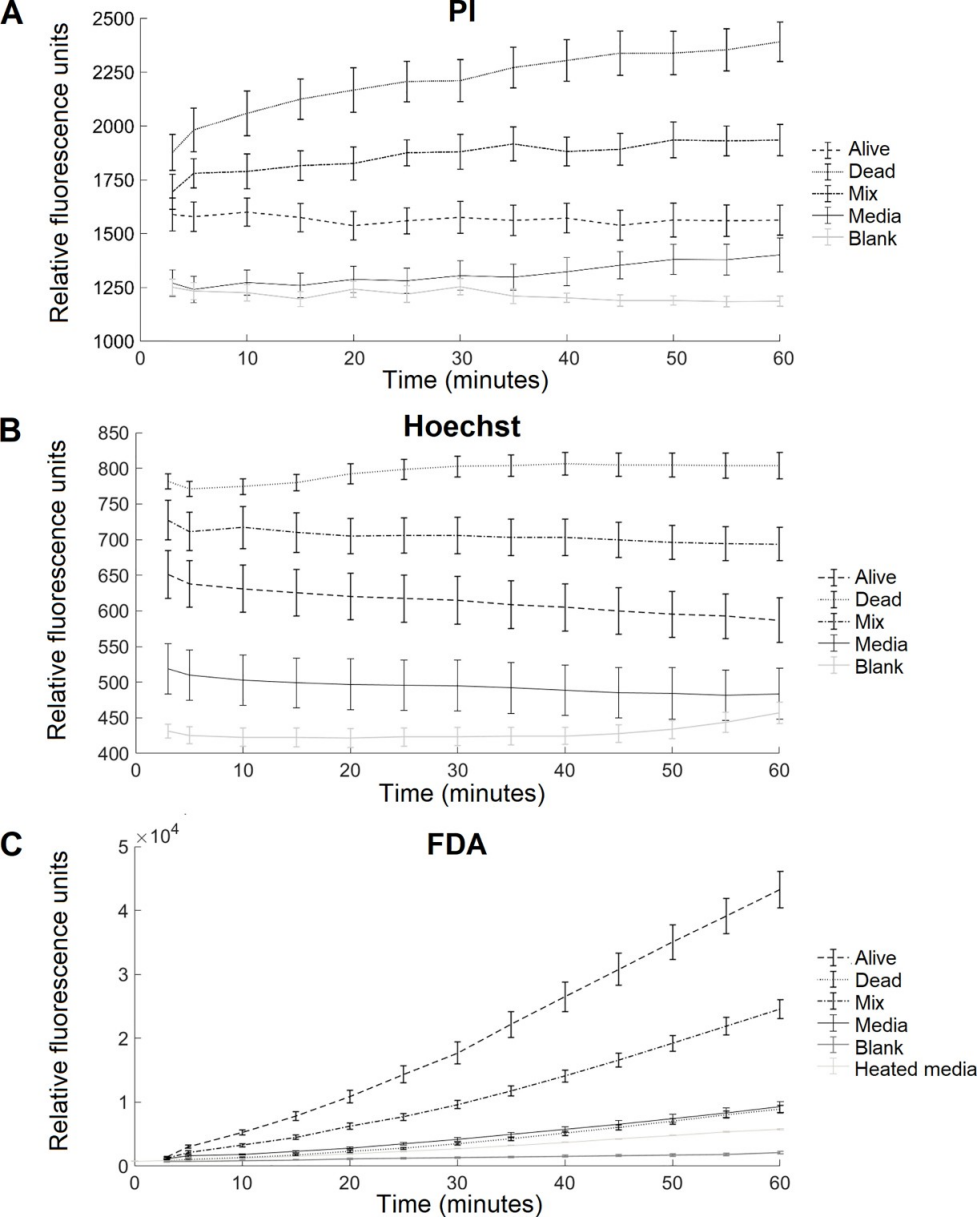
Fluorescence of PI (A), Hoechst (B) and FDA (C) stained cercariae over time. Each sample contains 100 ± 4 cercariae. Dead cercariae were heat-killed at 60°C for 5 minutes, and mixed samples contain 50% alive and 50% dead cercariae. Data points and error bars show the mean and SEM of nine wells (experiments run in triplicate and repeated three times).

PI-stained dead cercariae fluoresced the strongest, live cercariae the weakest, and mixed cercariae fluoresced evenly between live and dead samples (Fig 4A). The fluorescence of PI increased over time for dead and mixed samples, and remained relatively constant for live samples. These results are in line with results of Peak *et al*. [20] for schistosomula. The fluorescence of media and blank samples was significantly lower than that of cercaria samples. There was a statistically significant difference between PI fluorescence emissions of live, dead, and mixed samples (*p*<0.05 from 20 min, and *p*<0.01 from 50 min), and overall statistical significance increased over time. Therefore, an incubation time of 20 minutes was selected for PI experiments.

Similarly, Hoechst-stained dead cercariae fluoresced strongest, followed by mixed and live cercariae, as expected for a non-vital dye (Fig 4B). Media samples fluoresced significantly less, and the blank exhibited the lowest fluorescence. The post-hoc test shows that there is a significant difference (p<0.05) between all samples as of 20 minutes, yet the difference between alive and mixed samples never drops below a p-value of 0.01. The high standard error in the media, likely due to varying amounts of naturally fluorescing compounds released by cercariae and snails, is possibly the source of the error in the live and mixed samples.

FDA-stained live cercariae fluoresced very strongly, with mixed and dead samples fluorescing proportionately less (Fig 4C). The fluorescence of all cercaria samples increased significantly over time, though live cercariae showed the greatest increase. These also exhibit the highest standard error, potentially due to moving cercariae. Interestingly, the media (cercaria-free water) fluoresced more strongly than dead cercariae, indicating that the media contains stainable natural compounds such as proteins released by cercariae, or dissolved snail feces. To confirm this hypothesis, media was heated at 60°C for 5 minutes, which reduced fluorescence to that of the blank (Fig 4C). Overall, there is a significant difference between all cercaria samples after 5 minutes (p<0.05) and 10 minutes (p<0.01).

Based on these results, the fluorescence of Hoechst and PI samples should be read after 20 minutes incubation, and FDA samples after 5 minutes. For simplicity, all samples were read after 20 minutes (at which point alive, dead and mixed samples of all dyes still have a statistically significant difference of p<0.05).

### Determination of minimum parasite concentration

**Fig 5:**
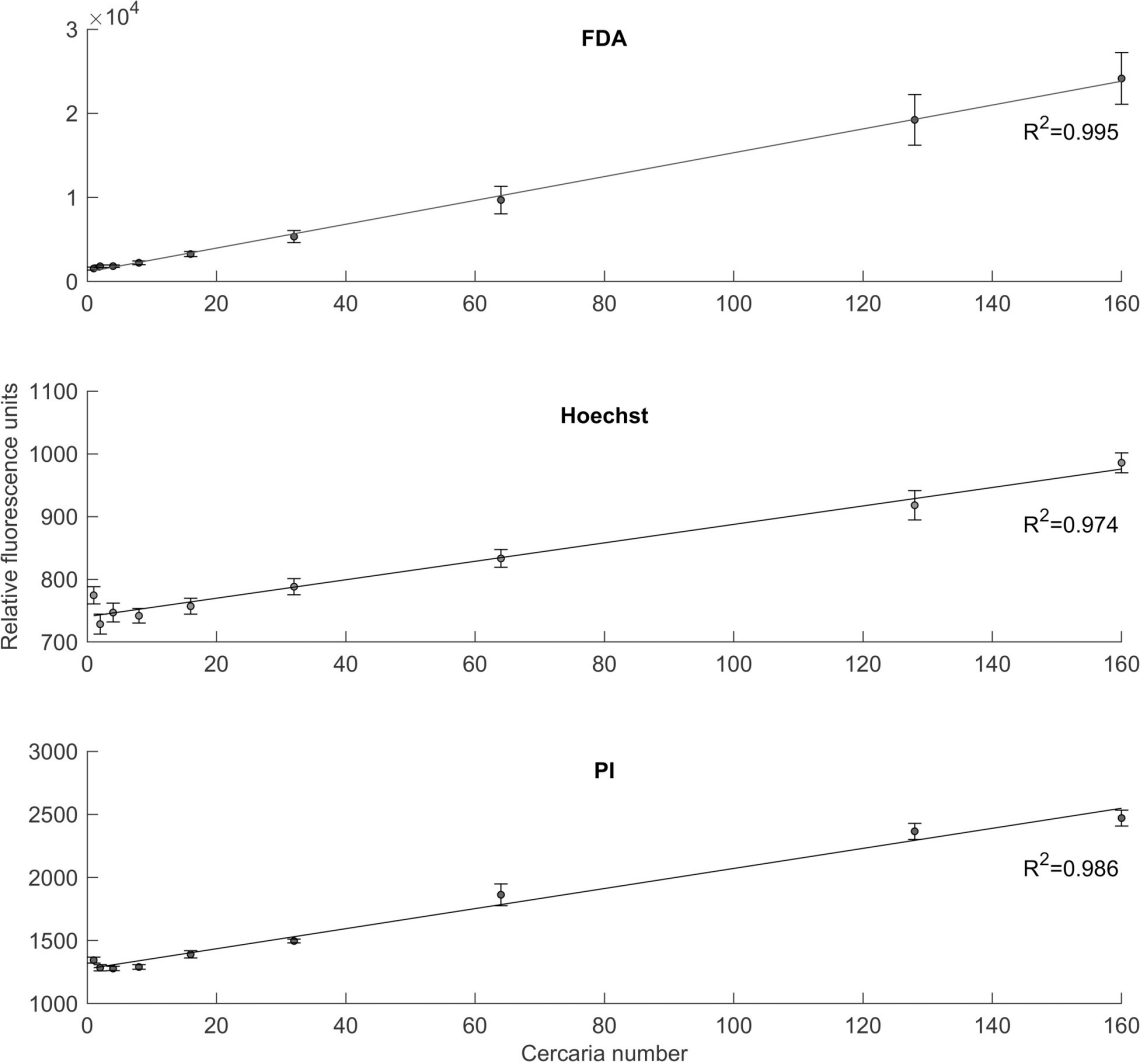
Fluorescence compared against cercaria number for the dyes considered in this study. Live (FDA) and dead (PI and Hoechst) cercariae were serially diluted from 128 to 1 cercaria/well, with an additional well containing 160 cercariae. Fluorescence measurements were taken at 20 minutes. Data points and error bars show the mean and SEM of nine wells (experiments run in triplicate and repeated three times).

To assess the sensitivity of the assays and to determine the correlation between number of cercariae and fluorescence, cercariae were serially diluted from 128 to 1 cercaria/well. An additional sample of 160 cercariae was prepared, as this was the maximum concentration of cercariae that could be continually achieved, given the number of snails available in the laboratory. Live cercariae were stained with FDA, and dead cercaria with Hoechst and PI. Overall, the results (Fig 5) show a strong linear correlation between cercaria numbers and fluorescence (R^2^ exceeding 0.974 for all dyes), and are in line with results found by the schistosomula-focused study by Peak *et al*. [20]. However, both PI and Hoechst samples exhibit non-linear relationships when containing fewer than 36 cercariae. The absolute minimum number of cercariae that should be used per well is therefore 36 parasites, however higher concentrations are recommended to ensure a more significant difference between fluorescence of live and dead samples. The required parasite concentration is significantly lower than found by Peak *et al*. [20] when testing schistosomula, potentially due to cercariae being larger in size.

### Quantifying cercarial viability using double-staining fluorescence assays

To determine if the two dual-stains can accurately measure the cercarial viability, and which combination of dual-stains is more accurate, samples with known proportions of live and dead cercariae were examined. Five viabilities ranging from 0% to 100% were tested (0%, 25%, 50%, 75%, 100% with ± 2% uncertainty), and these represent the “actual” viability. There is, however, uncertainty associated with these actual viabilities, resulting from uncertainty in cercaria numbers. The measured viability was calculated using fluorescence data collected at 20 minutes.

**Fig 6:**
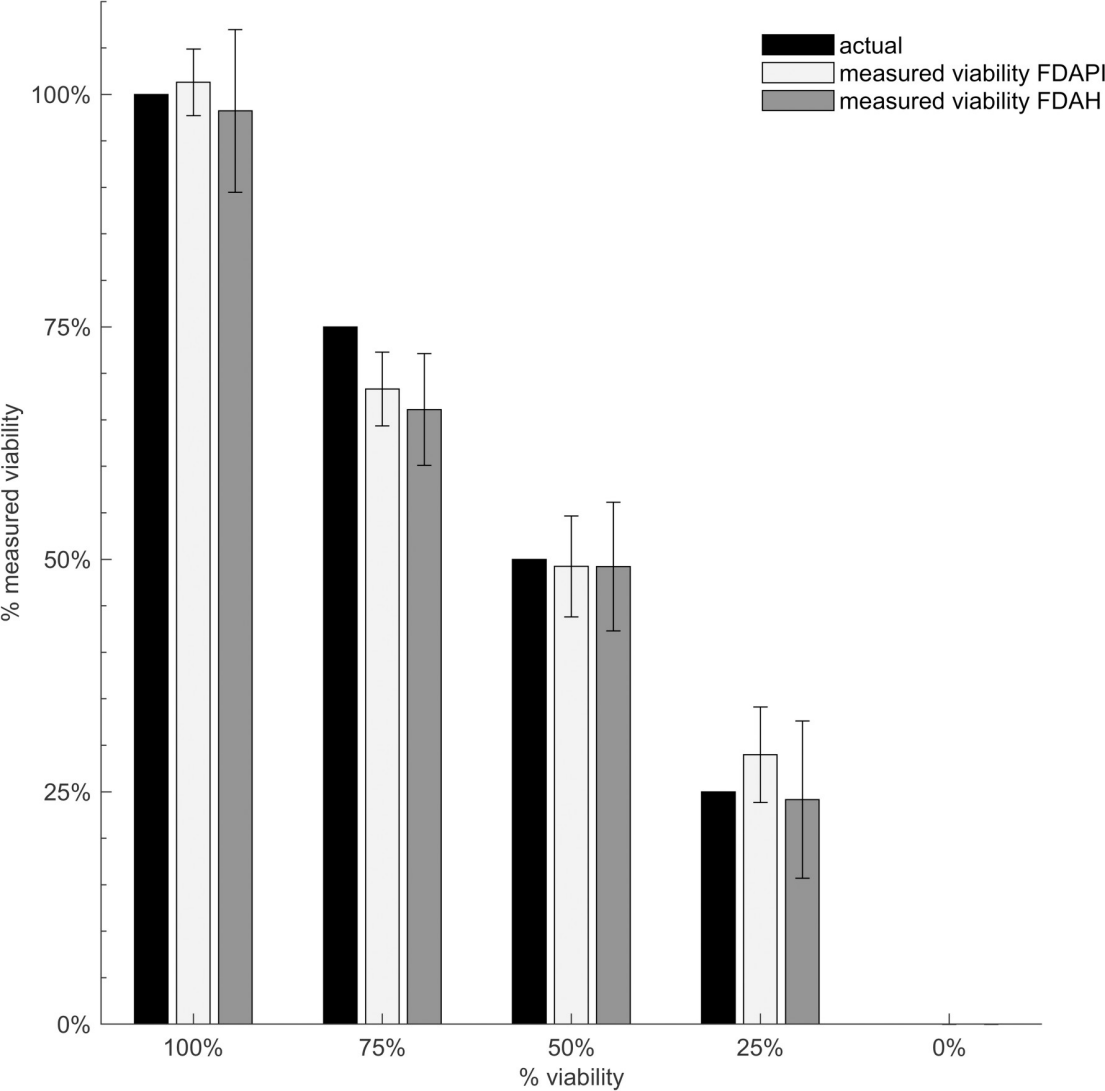
Measured versus actual viability of cercariae as determined by the dyes used in this study. Samples contained 100 ± 4 cercariae (ranging from 0–100% viable) and were stained with FDA-PI and FDA-H. Data points and error bars show the mean and SEM of nine wells (experiments run in triplicate and repeated three times). “Actual” viabilities are the known proportions of live and dead cercariae in each well, and “measured” viabilities are those quantified with the fluorescence data.

The assays could determine the viability with an average accuracy of 93.1% ± 5.5% (FDA-PI) and 92.2% ± 6.3% (FDA-H), as shown by Fig 6. FDA-H is less accurate and less precise, indicated by the higher standard errors. However, there is no statistically significant difference between the viabilities determined by the assays. FDA-H consistently underestimated the viability, potentially caused by inadequate assay conditions (e.g. incubation time or dye concentration).

### Comparison of microscopy and fluorescence assays as a means to quantify cercarial viability

A stereo-microscope was used to assess cercarial viability based on observations of movement, and the resultant data was compared to that collected using the fluorescence assays. Samples containing 30% viable cercariae (with an uncertainty of ± 2%) were tested for analysis by the two methods. The viability of 30% was randomly chosen. After the fluorescence of the samples was measured in the plate reader, three 50 μl aliquots were pipetted from each well onto a counting chamber and examined under the microscope. The number of viable and non-viable cercariae (in this case considered as moving and non-moving cercariae, respectively) was counted after gently shaking the chamber to promote movement. If cercariae did not move within five seconds, they were deemed non-viable along with non-moving separated cercariae (to avoid double counting, only separated heads were counted). All moving cercariae were classified as viable.

**Fig 7:**
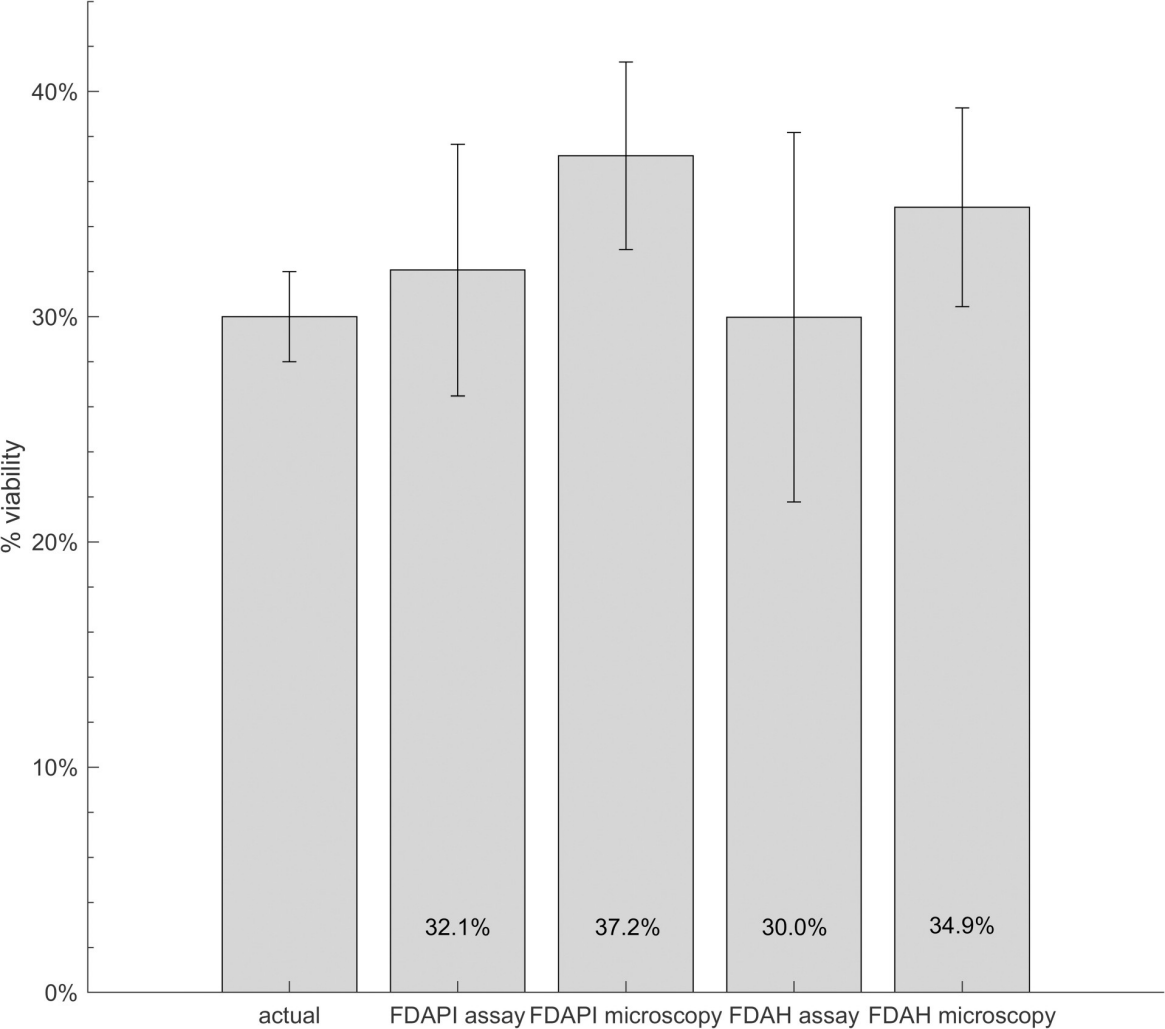
Comparing the viability quantified with fluorescence assays and microscopy. Samples containing 100 ± 4 cercariae with a viability of 30% ± 2% were stained with FDA-PI and FDA-H. Measurements were taken at 20 minutes. Three 50 μl aliquots of each sample were then examined under the microscope, and cercarial viability was recorded by counting moving (viable) and non-moving (non-viable) cercariae. Average quantified viabilities and SEMs are shown on the bar graph. The “actual” viability is the known proportion of live and dead cercariae in each well, chosen as 30%.

The results in Fig 7 show the average viabilities, quantified as 32.1% ± 5.6% and 30.0% ± 8.2% for FDA-PI and FDA-H assays, and 37.2% ± 4.2% and 34.9% ± 4.4% for the respective microscopy. There is no statistically significant difference between the measured viabilities. The fluorescence assays were on average more accurate (closer to the “actual” viability of 30% ± 2%) yet less precise, indicated by the higher standard errors. This is to be expected as the assays also analyze the media which increases the uncertainty in the data, unlike microscopy which only evaluates cercariae. The microscopy method overestimated the viability which indicates that cercariae were classified as viable when they were non-viable. However, without running host-infection experiments, it is difficult to know the exact viability of the sample.

## Discussion and conclusion

Here we test the use of two fluorescence assays for quantifying *S. mansoni* cercarial viability in a sample of water. The results indicate that both dual-stains, FDA-PI and FDA-Hoechst, can be used to accurately measure the viability of cercariae using a plate reader. To-date, microscopy has been the standard method for assessing viability. It examines cercarial movement and thereby classifies them as viable or non-viable. However, this method is labor-intensive, subjective, and not suited to high-throughput analysis. As we look towards eliminating schistosomiasis as a public health concern, more emphasis is being placed on water, sanitation and hygiene (WASH) as preventive measures. Water treatment plays a significant part in this, as access to safe, cercaria-free water for use in water contact activities such as bathing and laundry is linked to lower infection rates [33]. The lack of water treatment guidelines for schistosomiasis, as well as the lack of overall knowledge of how to make safe cercaria-free water, explains the need to test the effectiveness of water treatment processes. To facilitate this testing, a rapid, high-throughput and replicable method for assessing cercarial viability is needed, hence the motivation for this study.

To confirm that the three dyes used throughout these experiments exclusively stain live or dead cercariae, images of stained parasite larvae were taken on a fluorescence microscope. These showed some minor variation in cercaria fluorescence, which has also been observed in other studies [21,34]. This may be due to natural variation in dye-uptake, or differences in the focus or camera settings. To minimize variation, images were taken within 30 seconds after turning on the microscope, as long periods of excitation can lead to quenching and bleaching. However, these issues do not affect plate reader measurements.

Experiments examining parasite fluorescence over time confirm that live and dead cercariae fluoresce at significantly different intensities after short incubation times of 20 minutes. Furthermore, there is a strong correlation between number of cercariae and fluorescence. This study used 100 parasite larvae per well to ensure high sensitivity, however the results indicate that the assay may also be accurate for concentrations as low as 36 cercariae/well. Overall the results show that both assays could accurately quantify cercarial viability. FDA-H consistently underestimated the viability, suggesting that the procedure may need to be amended to reduce the measured proportion of dead cercariae (e.g. reducing the Hoechst concentration, or adjusting the plate reader gain or incubation time). Future studies could investigate the use of other dyes such as Hoechst 33342 or DAPI in place of Hoechst 33258, as these dyes might improve the accuracy of the assay. Hoechst 33342 has been shown to have increased cell-permeability compared to Hoechst 33258 [35,36], which could reduce the underestimation in viability caused by Hoechst 33258. Overall, there was no statistically significant difference between viability results of two fluorescence assays. As the procedures of the two assays are essentially identical, both the FDA-PI and FDA-H assays are recommended for the assessment of cercarial viability.

Differences between viabilities measured with microscopy and fluorescence assays may arise from separated cercariae. Tail loss has been shown to make the separated cercaria head (i.e. schistosomulum) more permeable and water intolerant than intact cercariae [37], potentially affecting dye uptake. The separation also causes cercariae to empty their acetabular gland and duct material [38], which could further affect staining. In microscopy, cercariae that have lost their tail but are still moving are classified as viable. In the fluorescence assay, however, the point of separation on the head and tail may already be stained with PI or Hoechst, as the cell membrane is ruptured and exposing DNA at this point, thereby decreasing the measured viability. Cercariae should only lose their tail if killed by the heat treatment, and hence be classified as non-viable in both assays. However, moving tail-less cercariae were observed under the microscope, potentially caused by pipetting or mixing the cercaria solution.

Standard errors were calculated in all experiments, likely arising due to varying cercaria numbers (calculated as ±4 cercariae per well) and the non-homogenous nature of samples. Samples containing live cercariae (e.g. live FDA- or Hoechst-stained cercariae) had especially high variances, possibly due to moving, or uneven distribution of cercariae. When observing live cercariae under the microscope, it was clear that they were attracted to the edge of the well. For this reason, the well plate was programmed to shake prior to each reading to excite the cercariae and encourage movement through the well, thereby increasing well-to-well precision [39]. Shaking must be kept constant, as it has been shown to influence the rate of hydrolysis of FDA [26]. In addition, the plate reader was set to measure at an orbital scan diameter of 4 mm, spaced between the center and side of the well. Other plate reader settings such as temperature must also be controlled as they can affect fluorescence readings. Although the plate reader was set to a constant temperature, inter-chamber temperature varied by ±0.2°C.

Standard errors may also be due to variations in the water media. Sometimes other microorganisms such as rotifers or paramecia were shed from snails and it was difficult to remove or control these. To minimize variations in the media, the number of snails and shedding time was controlled. Media was produced by filtering the cercaria water through a 20 μm mesh. Although this produced cercaria-free media, it also filtered out larger particles and organisms which contributed to fluorescence in unfiltered samples (alive, dead and mixed). It is difficult to get an exact fluorescence measurement of the water media, as this requires removing cercariae without removing other particles. Alternative methods for producing media should therefore be considered, potentially incubating non-infected snails in the same conditions as infected snails, and using this water to determine the fluorescence of the media. This will be especially relevant if testing real water samples and snails collected in the field, since these will likely carry other planktonic animals and be infected with non-human *Schistosoma* species. The use of the assays under these conditions will need to be tested.

To achieve a strong correlation between cercariae and fluorescence, it is important to test a high concentration of parasites. This also helps minimize the effect of variation in media measurements. A minimum of 36 larvae per well is recommended, which is very achievable using snails kept in the lab as well as infected snails collected in schistosomiasis-endemic regions. Lower concentrations did not present a linear correlation, potentially due to pipetting variability at such small volumes.

Although the three dyes are widely available and cheap, the assays require a high-sensitivity plate reader that can measure the fluorescence of non-homogeneous samples. Plate readers have a significant cost, and the additional requirement for black-sided microplates designed for fluorescence assays increases the overall cost of the assays. Some studies found that simpler, less advanced machines could not measure the fluorescence accurately enough when quantifying schistosomula viability [6,16]. The assays should therefore be verified for cercariae using less-advanced plate readers, e.g. for use in low-income laboratory settings.

The application of these dual-stains in water treatment experiments must also be tested. This study used heat-killed cercariae, however the accuracy must be validated for each water treatment process. For example, PI may not stain cercariae inactivated using ultra-violet (UV) disinfection, as the treatment inhibits proteins synthesis and modifies the surface membrane of cercariae, such as the concentration of glycocalyx that surrounds cell membranes [40,41]. UV may therefore not cause a breach in membrane which is required for staining. Furthermore, chemical (e.g. chlorination) or photochemical (e.g. UV disinfection) processes may interact with dyes and thereby affect results. The assays are limited to an incubation period of 20 minutes, which should be sensitive enough for the design of water treatment processes in less-developed areas which are unlikely to control the treatment processes on a greater level of accuracy.

Despite the requirement for advanced plate readers and microplates, these assays for quantifying viability of cercariae after water treatment are a significant improvement in terms of labor-time and subjectivity, when compared to microscopy. They will facilitate the research into effective water treatment processes against schistosome cercariae, and thereby help design much-needed safe water infrastructure for schistosomiasis-endemic regions.

## Supporting information

S1 Fig Hoechst-stained live cercariae. Images show the minimal labelling of live cercariae with Hoechst.
(TIF)

S2 Fig Hoechst-stained live and dead cercariae. Dead cercariae are strongly labelled with Hoechst, whereas live cercariae remain unstained (or minimally stained).
(TIF)
