# Supplementary figures and images for: Determining the viability of Schistosoma mansoni cercariae using fluorescence assays: An application for water treatment
Source: PLoS Negl Trop Dis. 2020 Mar 26;14(3):e0008176. doi: 10.1371/journal.pntd.0008176 (PMC7138324; doi:10.1371/journal.pntd.0008176)

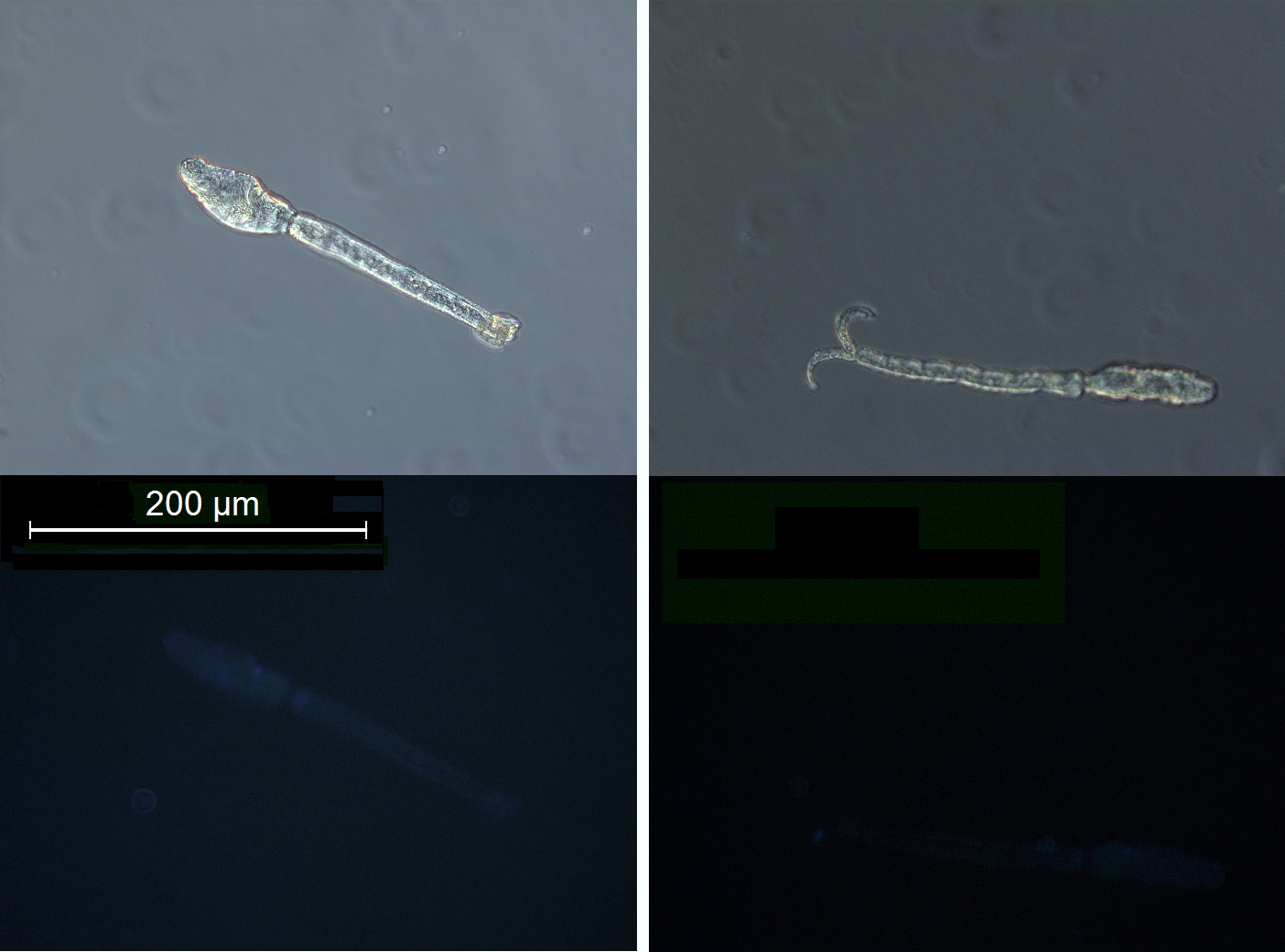

Supplement: S1 Fig — Images show the minimal labelling of live cercariae with Hoechst. (TIF) [file pntd.0008176.s001.tif]

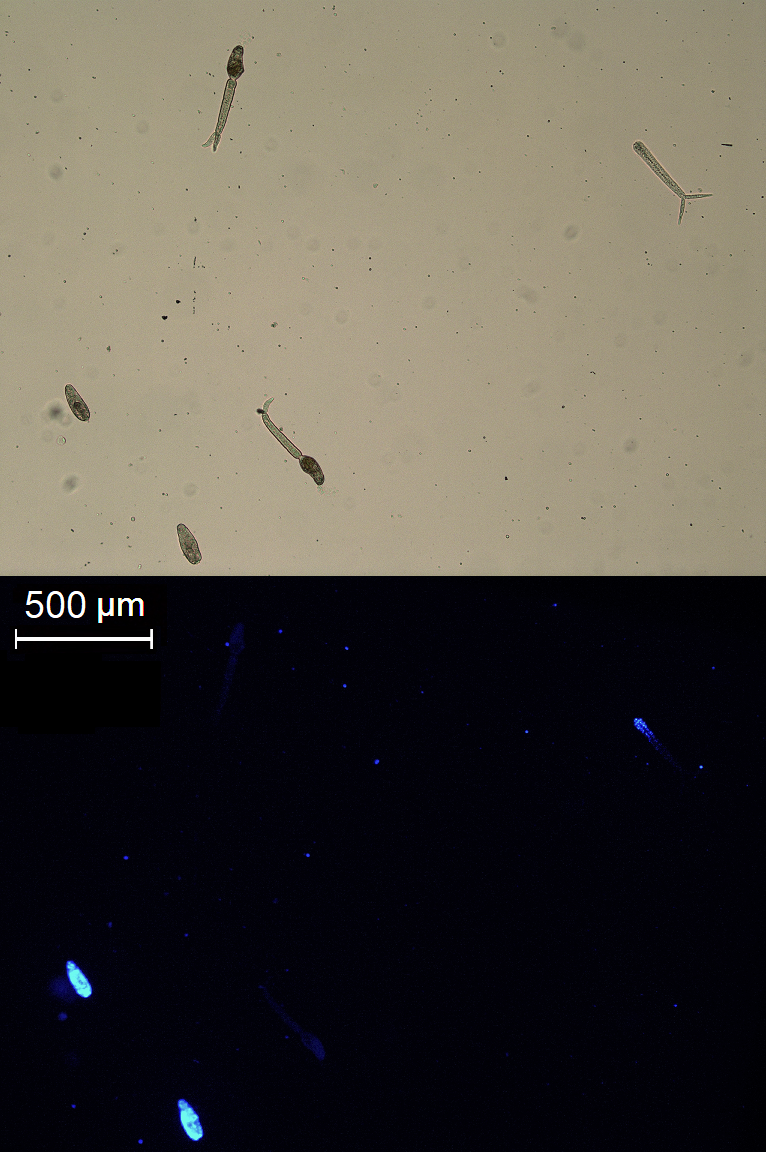

Supplement: S2 Fig — Dead cercariae are strongly labelled with Hoechst, whereas live cercariae remain unstained (or minimally stained). (TIF) [file pntd.0008176.s002.tif]
